# Cervical cancer-derived exosomal miR-663b promotes angiogenesis by inhibiting vinculin expression in vascular endothelial cells

**DOI:** 10.1186/s12935-021-02379-9

**Published:** 2021-12-19

**Authors:** Xuewu You, Wenxiong Sun, Ying Wang, Xiaoli Liu, Aihong Wang, Lu Liu, Sai Han, Yu Sun, Junhua Zhang, Lingyu Guo, Youzhong Zhang

**Affiliations:** 1 Department of Obstetrics and Gynecology, Qilu Hospital, Cheeloo College of Medicine, Shandong University, 107 Wenhua Xi Road, Jinan, 250012 Shandong People’s Republic of China; 2 Department of Obstetrics and Gynecology, Peking University People’s Hospital, Beijing, 100044 People’s Republic of China; 3 Department of Obstetrics and Gynecology, Yidu Central Hospital of Weifang, Weifang, 262500 Shandong People’s Republic of China; 4 Department of Obstetrics and Gynecology, Feicheng Hospital Affiliated to Shandong First Medical University, Taian, 271600 Shandong People’s Republic of China

## Abstract

**Background:**

Angiogenesis provides essential nutrients and oxygen for tumor growth and has become the main mechanism of tumor invasion and metastasis. Exosomes are nanoscale membrane vesicles containing proteins, lipids, mRNA and microRNA (miRNA), which mediate intercellular communication and play an important role in tumor progression. Accumulated evidence indicates that tumor-derived exosomal miRNAs participate in the tumor microenvironment and promote angiogenesis.

**Methods:**

Bioinformatic target prediction and dual luciferase reporter assays were performed to identify the binding site between miR-663b and the 3′-UTR of vinculin (VCL). VCL overexpression lentivirus and miR-663b overexpression/inhibition lentivirus were used to create a VCL overexpression model and miR-663b overexpression/inhibition model in-vitro. Immunohistochemistry (IHC) assays and western blot assays were used to detect protein expression. Exosome-cell cocultures, wound healing assays, tube formation assays and transwell assays were used to measure the migration and tube formation ability of vascular endothelial cells [human umbilical vein endothelial cells (HUVECs)]. siRNA targeted VCL was used to knockdown VCL.

**Results:**

In the present study, we found that miR-663b was elevated in cervical cancer tissue and exosomes. miR-663b could bind the 3′-UTR of VCL and inhibit its expression. VCL is downregulated in cervical cancer, and decreased VCL has a negative correlation with a high level of miR-663b. Further studies demonstrated that exosomes secreted by cervical cancer cells can deliver miR-663b to HUVECs and inhibit the expression of VCL, thereby promoting angiogenesis and tumor growth.

**Conclusions:**

miR-663b derived from cancer cell exosomes acts as a driving factor for angiogenesis and a potential target of antiangiogenic therapy in cervical cancer. Our findings illustrated a new signaling pathway, including exosomes, miRNAs and target genes, which provides potential targets for antiangiogenic therapy.

## Introduction

Cervical cancer remains an important cancer worldwide and was responsible for an estimated 570,000 cases and 311,000 deaths in 2018 worldwide, making it the fourth most frequently diagnosed cancer and the fourth leading cause of cancer death in women [1]. Although morbidity and mortality have been declining due to screening detection and new treatment strategies, there is still an urgent need to explore new effective treatment targets for patients who are not suitable for surgery or in whom the effects of radiotherapy and chemotherapy are not obvious [2]. Previous studies have shown that tumor growth will not exceed 1–2 mm or metastasize to other organs due to insufficient oxygen and nutrient supply if there is no new blood vessel formation [3, 4]. Angiogenesis is essential for tumor proliferation, migration and invasion and is also a very common phenomenon in cervical cancer [5, 6]. Anti-angiogenic monoclonal antibody drugs such as Bevacizumab combined with chemotherapy can prolong the overall survival of cervical cancer patients compared with intravenous chemotherapy alone [7]. Therefore, it is essential to suppress the progression of cervical cancer by exploring new antiangiogenic molecular mechanisms of angiogenesis.

Intercellular communication between host malignant cells and stromal cells is necessary in the tumor microenvironment (TME) [8]. Exosomes are 30–200 nm extracellular vesicles (EVs) composed of lipid bilayers, which can transport a variety of functional biomolecules, including proteins, microRNAs (miRNAs) and DNA, to new target cells to mediate signal transduction and cell-to-cell communication. Previous studies have reported that exosomes released from different cells have various characteristics and are widely distributed in blood, saliva, urine, and milk to perform related functions [9, 10]. miRNAs are small noncoding RNAs with a length of approximately 22 nt. By completely or incompletely binding to the 3'-UTRof target mRNA, miRNA can cause mRNA degradation or posttranscriptional suppression [11]. Recently, accumulated evidence has shown that cancer-derived exosomes play a crucial role in tumor occurrence, progression, metastasis, drug resistance and angiogenesis [12–16]. Therefore, strategies to control the loading, transportation or uptake of exosomal miRNA may be effective for the treatment of cervical cancer.

Vinculin (VCL) is a widely expressed actin binding protein that plays a key role in focal adhesion formation [17], cell proliferation [18] and regulation of the actin cytoskeleton [19, 20]. Recent studies have shown that the expression of VCL protein is decreased in squamous carcinoma [21, 22], nonsmall lung cancer [23], breast cancer [24] and other malignant tumors and is related to the enhanced invasion, metastasis and apoptosis of cancers [25–27]. However, the biological role and value of VCL in cervical cancer have not been fully studied, which suggests that many studies need to be carried out to clarify its mechanism.

In our present study, we found that miR-663b was significantly upregulated in cervical cancer tissue and exosomes secreted from cervical cancer cell lines. Moreover, the level of miR-663b had a negative correlation with VCL protein, which was significantly downregulated in cervical cancer tissues. Exosomes derived from CaSki cells contain miR-663b and transport it to human umbilical vein endothelial cells (HUVECs), resulting in decreased expression of VCL protein and enhanced migration and tube formation. Bioinformatics analysis and dual luciferase analysis proved that miR-663b directly targets the 3′-UTR of VCL mRNA. Moreover, we found that knocking down the VCL protein enhanced the migration and tube-formation ability of HUVECs, while overexpression of VCL had the opposite effect. In-vivo studies have shown that exosomes containing miR-663b significantly promote the growth and angiogenesis of tumors implanted in mice. Therefore, our research reveals a new molecular mechanism of angiogenesis in cervical cancer.

## Methods

### Tissue collection

Fifty-nine pairs of cervical cancer tissues and normal cervix were collected from patients during surgery at Qilu Hospital of Shandong University from June 2017 to December 2020. Written informed consent was obtained from all subjects (KTLL-2017-560). The tissues were stored at − 80 °C until required for RNA and protein extraction.

### Cell culture

HEK 293 T cells and the human cervical cell lines SiHa, HeLa, CaSki, and H8 were obtained from the Cancer Center Laboratory of Shandong University. HEK 293T, HeLa and H8 cells were cultured in DMEM (Gibco, CA, USA), SiHa cells were cultured in MEM (Gibco, CA, USA) and CaSki cells were cultured in RPMI-1640 (Gibco, CA, USA) medium supplemented with 10% fetal bovine serum (FBS, Biological Industries, CT, USA). Primary HUVECs were isolated from the umbilical cord of a fetus obtained by cesarean section. Sterile PBS was used to clean blood stains on the surface of the umbilical cord. One end of the umbilical cord was ligated with a sterile cotton thread, and the other end of the umbilical vein was injected with type IV collagenase (Solarbio, Beijing, China, C8160) by a gavage needle and a 5-ml syringe. Then, the umbilical cord was ligated and placed in an incubator at 37 °C for 30 min and gently rubbed every 10 min. Finally, we collected the collagenase-HUVEC mixture from the umbilical vein and washed it twice with PBS. The mixture was transferred into a 15-ml centrifuge tube, and the digestion was terminated by an equal volume of complete medium. After centrifugation at 800 rpm for 5 min, the cell pellet was resuspended in endothelial cell culture medium containing 10% serum and inoculated into a petri dish. All cell lines were cultured at 37 °C in a moistened air atmosphere containing 5% CO_2_.

### Exosomes isolation

Exosomes were isolated from culture medium by serial centrifugation. Briefly, cells were cultured in exosome-free FBS (VivaCell, Shanghai, China), and the medium was collected after 48 h. Cells or other debris were removed by centrifugation at 300*g* for 10 min and 2000*g* for 10 min, and then secondary centrifugation at 10,000*g* for 20 min was performed to remove other larger vesicles. Next, the medium was ultracentrifuged at 110,000*g* for 70 min, washed with PBS and centrifuged at 110,000*g* for another 70 min. The exosome pellet was resuspended in PBS at an appropriate volume. A BCA protein assay kit (Beyotime, China) was used to measure the concentration of exosome suspension.

### Nanoparticle tracking analysis

The number and size distribution of isolated exosomes were measured by a ZetaView PMX120 instrument (Particle Metrix, Bavaria, Germany) through a 1-ml syringe at room temperature. The results were analyzed by the corresponding software ZetaView 8.02.28 and calculated on average three times.

### Transmission electron microscopy (TEM)

Isolated exosomes were fixed with 2% paraformaldehyde for 5 min and then dropped onto a Formvar copper carbonate grid with glow discharge for 1 min. Next, 2% uranyl acetate was used to negatively stain the cells for another 1 min. After sample drying, we used an HT7800 electron microscope (Hitachi, Tokyo, Japan) to photograph the grid with an acceleration voltage of 80 kV.

### PKH67 staining for exosomes

Isolated exosomes were fluorescently labeled using PKH67 green Fluorescent Cell Linker Kits (Sigma, MO, USA) according to the manufacturer’s instructions. Exosomes were incubated with 4 µl of PKH67 and 1 ml of diluent C separately for 5 min at room temperature. One milliliter of 5% bovine serum albumin (Solarbio, Beijing, China) was added to stop staining. The mixture was centrifuged at 110,000*g* for 2 h at 4 °C. After washing with PBS, pure PKH67-labeled exosomes were obtained by centrifugation at 110,000*g* for another 2 h. Labeled exosomes were then resuspended in complete medium and incubated with CaSki cells at 37 °C for 0 h, 2 h, 4 h and 8 h. A laser confocal microscope (LSM880, Zeiss, Jena, Germany) was used to visualize the incorporation of exosomes into CaSki cells.

### Immunohistochemical staining and evaluation

The tissues were fixed in 4% paraformaldehyde, embedded in paraffin and cut into 4‐μm slices. Endogenous peroxidase was blocked by hydrogen peroxide. Antigen retrieval was performed by heating in the microwave on band 3 for 10‑15 min (700 W oven). Subsequently, the slices were incubated with anti-vinculin antibody (1:1000; CST, 13901 T) and anti-CD31 antibody (1:1000, Abcam, ab28464) overnight at 4 °C for 18 h. Finally, the slices were stained with DAB (DAB Kit, ZSGB-BIO, China) and crystal violet at room temperature. PBS without primary antibody was used as a negative control. The slices were observed and photographed in at least three sections by a JEM‑1200 EX II electron microscope (JEOL, Tokyo, Japan).

### miRNA prediction and dual luciferase reporter assay

We used miRWalk (http://mirwalk.umm.uni-heidelberg.de/), miRMap (https://mirmap.ezlab.org/) and TargetScan (http://www.targetscan.org/) to predict the upstream miRNAs of VCL. A dual luciferase reporter assay was performed to prove the regulation of miR-663b and VCL in 293 T cells. Wild-type (WT) and mutant (MUT) VCL 3′-UTRs were synthesized and inserted into the pmiR-REPORT luciferase plasmid (OBIO, Shanghai, China). Then, pmiR-REPORT Luciferase-VCL 3′UTR (WT), pmiR-REPORT Luciferase-VCL 3′UTR (MUT) and pRL-CMV (CON) (Promega, WI, USA) were cotransfected into 293T cells with miR-663b mimics or NC (GenePharma, Shanghai, China). After 48 h of transfection in a 96-well plate, a dual fluorescence luciferase reporter gene detection system (Promega, WI, USA) was used to detect fluorescence. The experiments were normalized to Renilla luciferase activity.

### RNA isolation and quantitative real‑time transcription‑polymerase chain reaction (qRT-PCR)

Total RNA was extracted from the cells and exosomes using TRIzol reagent (Invitrogen, Thermo Fisher Scientific Inc. USA), and the concentration was detected by a spectrophotometer (Thermo Fisher Scientific Inc., MA, USA). cDNA was prepared using M-MLV Reverse Transcriptase (Invitrogen, CA, USA, 1175950) for RT-PCR. For analysis of miRNA expression, qPCR was performed by using SYBR (TaKaRa, Kyoto, Japan) and carried out on a StepOne™ PCR amplifier (Applied Biosystems, USA). Final data were normalized to U6 and calculated through the 2^−ΔΔ^CT method. The primer sequences for miRNA were purchased from GenePharma (Shanghai, China) and are listed as follows:

miR-663b: F: 5′-GGTGGCCCGGCCGTGC-3′

R: 5′-TATCCTTGTTGACGACTCCTTGAC-3′

U6: F: 5′-CAGCACATATACTAAAATTGGAACG-3′

R: 5′-ACGAATTTGCGTGTCATCC-3′

### Western blot analysis

Total proteins extracted from cells, exosomes or tissues were lysed with RIPA buffer and quantified by a BCA Protein Assay Kit (Beyotime, Beijing, China). Protein samples (30 µg/well) were loaded and separated on a 12% polyacrylamide gel and then transferred to a polyvinylidene fluoride membrane. After blocking with 5% nonfat milk in PBS solution at room temperature for 1 h, membranes were then incubated overnight with the following primary antibodies at 4 °C: anti-CD63 (1:1000, Abcam, CA, UK, ab59479), anti-TSG101 (1:1000, Abcam, CA, UK, ab125011), anti-CD81 (1:2000, Abcam, CA, USA, ab109201), anti-GAPDH (1:1000, CST, MA, USA, #5174), anti-VCL (1:1000, CST, MA, USA, #13901), and anti-CD31 (1:1000, CST, MA, USA, #13116). Subsequently, membranes were washed with Tris-buffered saline with Tween-20 and incubated with HRP-conjugated antibody (1:1000, CST, MA, USA) at room temperature for 2 h. The blots were visualized with an ECL substrate kit (Thermo Fisher Scientific Inc., MA, USA) and quantified by ImageJ software (v.8.0, National Institutes of Health, USA).

### Wound healing assay

HUVECs were seeded in 6-well plates and cultured in complete medium until a monolayer formed. Then, a straight wound was generated with a sterile 100 µl pipette by quickly scratching the confluent monolayer of cells. The floating cells were washed with PBS, and the medium containing 3% FBS was replaced to culture the remaining cells. Images of the scratches were taken by a JEM-1200 EX II electron microscope (JEOL, Tokyo, Japan) at 0 h and 12 h after scratching. Wound closure was evaluated by ImageJ software (v.8.0, National Institutes of Health, USA).

### Migration assays

HUVECs (3 × 10^4^) from different treatment groups were suspended in 200 μl of serum-free medium and seeded in upper Transwell chambers (8 μm pore size; Corning Costar, MA, USA). The lower chamber was filled with 600 μl of medium with 10% FBS. Cells were allowed to migrate at 37 °C and 5% CO_2_ for 18 h and then harvested by the following steps. The nonmigratory cells on the upper surface of the membrane were wiped off with a cotton tip, and the migrated cells attached to the lower surface were fixed in methanol for 15 min and stained with crystal violet (Beyotime, Beijing, China) for 15 min. After that, 3–5 random fields of view were selected for photography under a JEM-1200 EX II electron microscope (JEOL, Tokyo, Japan).

### Tube formation assay

Ninety-six-well plates coated with Matrigel (BD Biosciences; 50 μl/well) were placed at 37 °C for 30 min to solidify. Then, 3 × 10^3^ HUVECs from different treatment groups were suspended in 100 μl of 3% FBS-containing medium and seeded in solid Matrigel. After incubation at 37 °C for 4 h, HUVECs formed a tubular structure and were imaged using a JEM-1200 EX II electron microscope (JEOL, Tokyo, Japan). Tube formation was evaluated by ImageJ software (v.8.0, National Institutes of Health, USA).

### Plasmid construction, RNA interference and transfection assay

For miR-663b upregulation and downregulation, the lentiviral vector hU6-MCS-Ubiquitin-EGFP-IRES-puromycin designed by GenePharma Co., Ltd. (Shanghai, China) was used in our study. VCL-overexpressing plasmids were designed by He Yuan Co., Ltd. (Shanghai, China). The lentiviral vector PSLenti-EF1-EGFP-P2A-Puro-CMV-MCS-3xFLAG-WPRE was used to improve the transduction efficiency. After 14 days of selective culture with puromycin dihydrochloride (2 μg/ml; Amresco, Solon, OH, USA), CaSki cell lines stably expressing miR-663b and HUVEC cell lines stably expressing VCL were used for subsequent experiments. Small interfering RNA (siRNA) targeting VCL was synthesized by GenePharma Co., Ltd. (Shanghai, China). Cell transfection was performed using Lipofectamine 2000 (Invitrogen, CA, USA) and Opti-MEM (Gibco, CA, USA). After transfection in Opti-MEM for 6 h, the complete culture medium was replaced, and the cells were cultured for the following experiments. Total RNA and total protein were isolated from the cells 24 h or 48 h after transfection for qPCR analysis and Western blot analysis. For convenience, we abbreviated the overexpressing cell lines as HUVEC-VCL and the inhibition of VCL as si-VCL, while the control cell lines were abbreviated as HUVEC-NC and si-NC.

### Mouse xenograft model

Five-week-old female athymic nude mice were purchased from Weitong Lihua Biotechnology Co., Ltd. (Beijing, China) and approved by the Institutional Animal Research Ethics Committee of Shandong University. Mice were randomly divided into five groups (6 mice/group) and injected with 5 × 10^6^ CaSki cells subcutaneously. After growing to a tumor size 50 mm^3^ for 15 days, 10 µg of exosomes resuspended in 20 µl of PBS was injected into the center of each tumor every 3 days. The tumor volumes were examined every 3 days and calculated using equation V = A × B^2^/2(mm^3^). (A: the largest diameter; B: the perpendicular diameter). After 15 days, primary tumors that reached a volume of approximately 500 mm^3^ were excised for subsequent experiments.

### Statistical analysis

Data are expressed as the median values ± standard deviation (SD) of each group using GraphPad Prism 8 software. All experiments were performed at least in triplicate. Comparisons between the groups were analyzed by Student’s *t* test (unpaired, two-tailed) or one-way analysis of variance (ANOVA). Statistical significance was set at p < 0.05.

## Results

### VCL is downregulated in cervical cancer

**Fig. 1:**
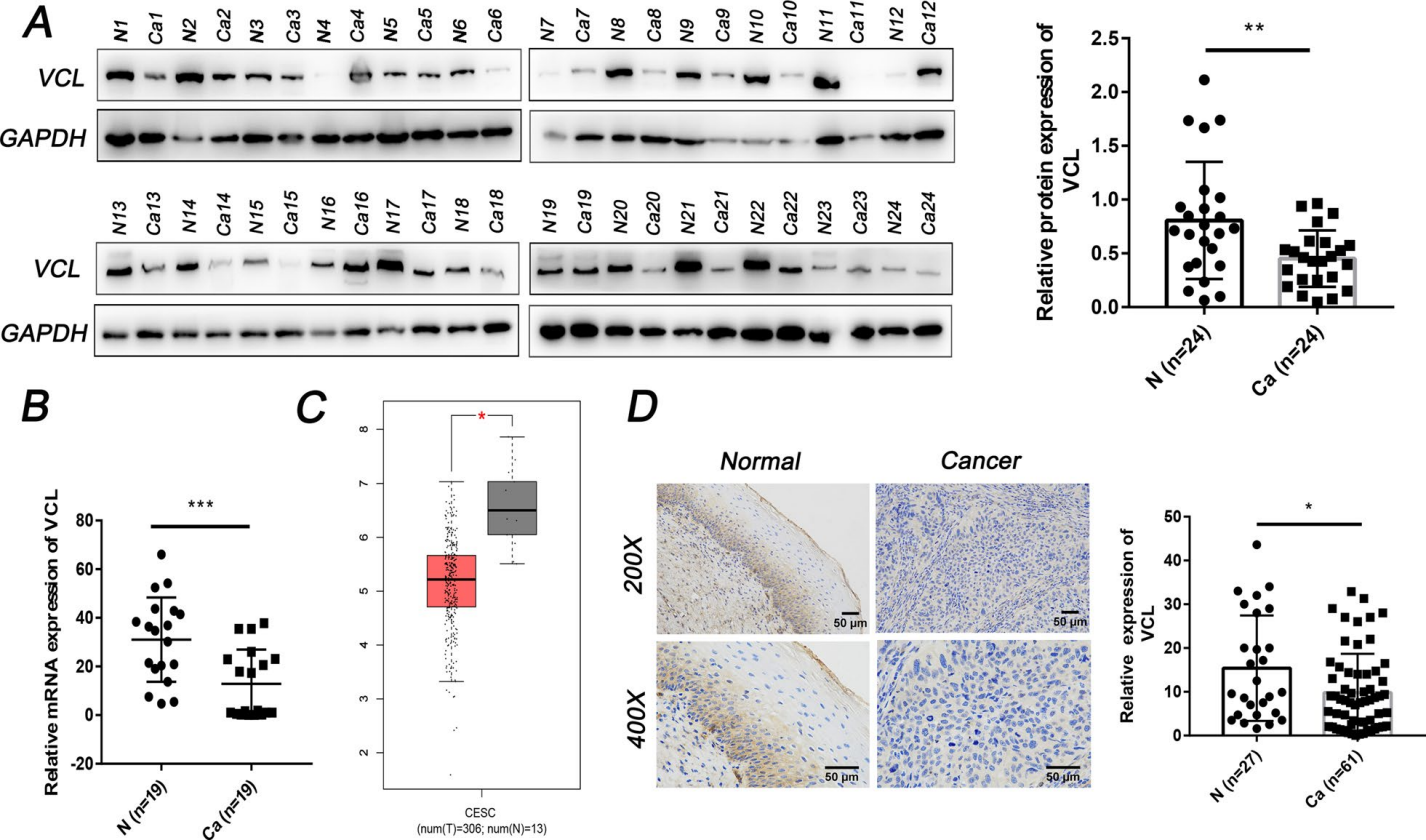
VCL is downregulated in cervical cancer. **A** Protein expression of VCL in 24 normal cervical tissues and 24 tumor tissues was determined by Western blot analysis. **B** The mRNA level of VCL in 19 normal cervical tissues and 19 tumor tissues was determined by qPCR. **C** Expression levels of VCL in cervical cancer tissues from TCGA datasets. **D** The expression of VCL in normal cervix and cervical cancer tissues was determined by IHC (×100 and ×200). *P < 0.05, **P < 0.01, ***P < 0.001. N, normal cervix; Ca: cervical cancer; VCL, vinculin

We explored the expression of VCL in cervical cancer from clinical samples. WB and PCR results showed that the protein and mRNA levels of VCL in cervical cancer tissue were significantly lower than those in normal cervix (Fig. 1A, B). The TCGA database (http://gepia.cancer-pku.cn/detail.php?gene=VCL) also revealed decreased expression of VCL in cervical cancer tissues (Fig. 1C). IHC analysis of 88 patients was performed to explore the distribution of VCL in cervical cancer, and the results showed that VCL was downregulated in cancer tissue compared with normal cervix (Fig. 1D). These results indicated that VCL may act as an anticancer gene in cervical cancer.

### VCL is the direct target of miR-663b

**Fig. 2:**
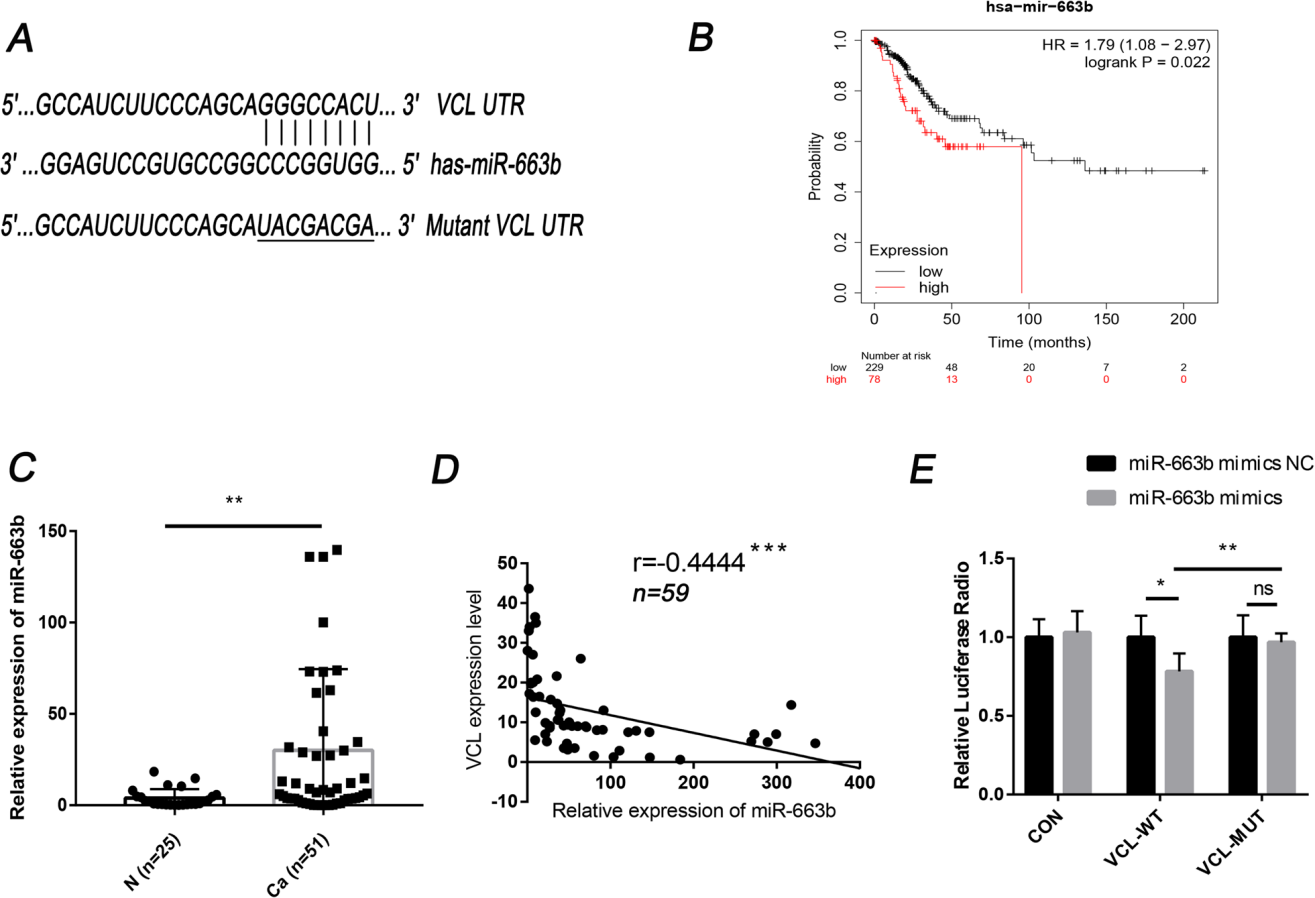
VCL is the direct target of miR-663b. **A** The putative binding site between VCL and miR-663b. **B** The survival curve of cervical cancer associated with miR-663b levels. **C** miR-663b expression levels in 25 normal cervical tissues and 51 tumor tissues were determined by qPCR. **D** The correlations between the expression of VCL and miR-663b. **E** Luciferase activity was detected in 293T cells cotransfected with miR-663b mimics or miRNA NC and WT-VCL or MUT-VCL or empty vector. *P < 0.05, **P < 0.01, ***P < 0.001. VCL, vinculin; CON, control; WT, wild-type; MUT, mutant

Bioinformatics tools (TargetScan, miRMap, and miRWalk) were applied to screen the potential regulation upstream of VCL. Among all miRNAs that may be associated with VCL, we found that miR-663b was upregulated in various cancers and had a 7 bp binding site with the VCL mRNA 3′-UTR (Fig. 2A). Then, we used the Kaplan–Meier plotter database (https://kmplot.com/analysis/index.php?p=service) to predict and analyze the effect of miR-663b on the prognosis of cervical cancer. The results showed that the survival rate of the miR-663b-high expression group (n = 229) was lower than that of the miR-663b-low expression group (n = 78) with hazard ratio (HR) 1.79 and 95% confidence intervals (CI) 1.08–2.97 (P = 0.022) (Fig. 2B). qPCR was then performed to detect the expression level of miR-663b in 51 cervical cancer tissues and 25 normal tissues. The results showed that the expression of miR-663b was increased in cervical cancer tissues (Fig. 2C). Based on these results, we inferred that miR-663b may act as an oncogene in cervical cancer. To further determine the relationship between miR-663b and VCL, a correlation analysis between miR-663b and VCL protein levels was conducted, and the results showed that there was a negative correlation between VCL and miR-663b (Fig. 2D).

Next, we further investigated the regulatory relationship between miR-663b and VCL by dual luciferase reporter assay. Synthesized wild-type and mutant VCL 3′-UTRs were inserted into the luciferase reporter plasmid and combined with miRNA mimics (miR-663b mimics) and scrambled negative control RNA (miR-663b mimics NC) to transfect 293 T cells. The results showed that, compared with cells transfected with control RNA, the overexpression of miR-663b caused decreased luciferase activity in wild-type cells but hardly affected the luciferase activity in mutant cells (Fig. 2E). These results indicated that miR-663b directly targets VCL and inhibits its expression.

### Exosomes transport miR-663b to HUVECs and inhibit VCL expression

**Fig. 3:**
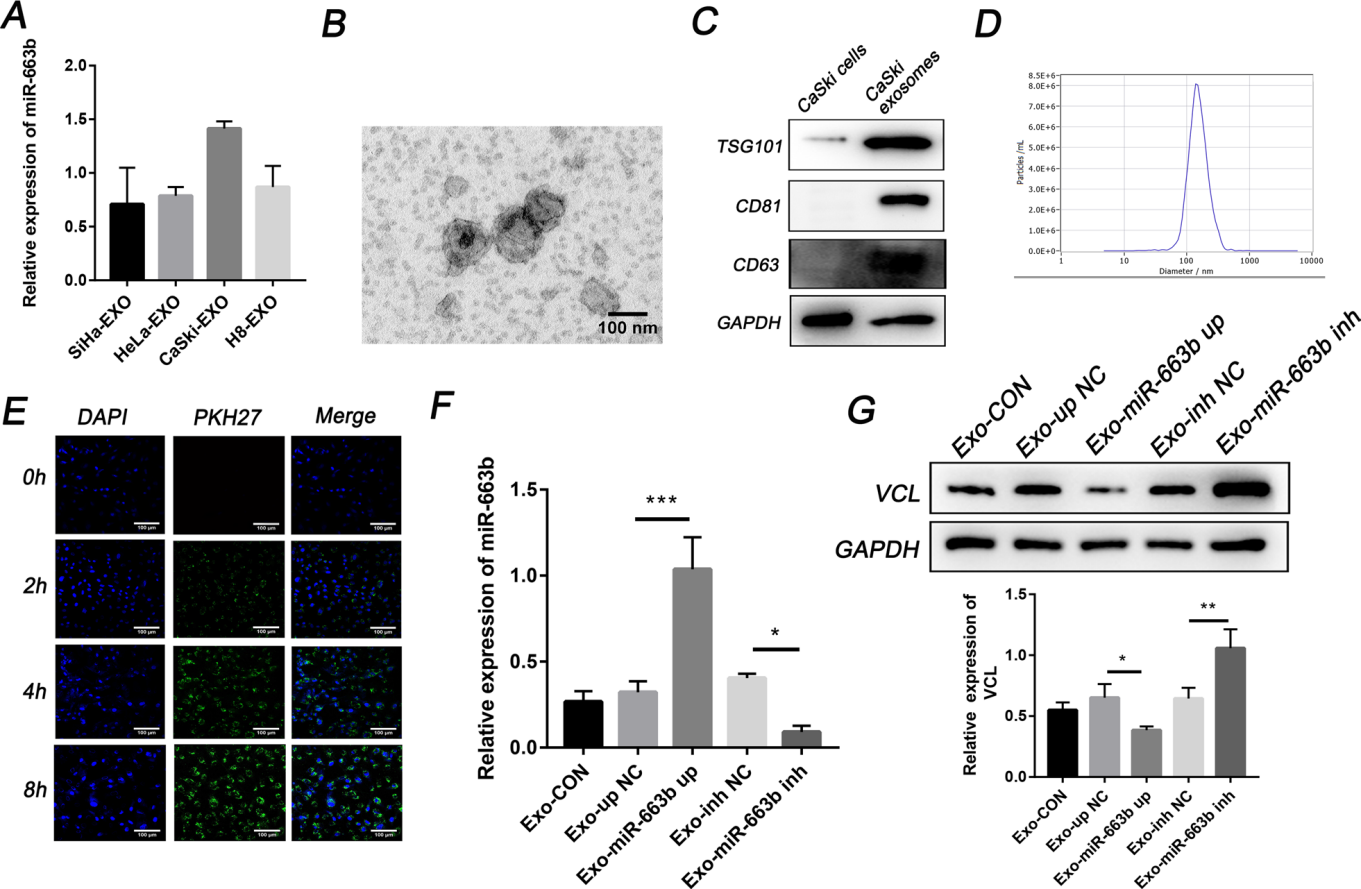
Exosomes transport miR-663b to HUVECs and inhibit VCL expression. **A** qPCR analysis of the relative expression of miR-663b in exosomes harvested from SiHa, HeLa, CaSki and H8 cell lines. **B** TEM images of exosomes isolated from CaSki cells. **C** CaSki cell-secreted exosome-positive markers CD63, TSG101, CD81 and GAPDH were detected by Western blot analysis. **D** Nanoparticle size analysis of CaSki cell-secreted exosomes. **E** CaSki cells pretreated with PKH67-labeled exosomes for 0, 2, 4 and 8 h were stained with DAPI (blue) for confocal microscopy analysis (magnification ×200). **F** qRT-PCR assay of miR-663b in HUVECs pretreated with CaSki exosomes, miR-663b-up exosomes, miR-663b-inhibitor exosomes and NC exosomes. **G** VCL expression in HUVECs treated with CaSki exosomes, miR-663b-up exosomes, miR-663b-inhibitor exosomes and NC exosomes and the corresponding quantitative analysis. *P < 0.05, **P < 0.01 and ***P < 0.001. VCL, vinculin; TEM, transmission electron microscopy; TSG101, tumor susceptibility gene 101

Previous studies have reported that exosomes can carry small molecules such as miRNA to mediate cell-to-cell communication between different types of cells, thereby promoting tumor progression [28]. Therefore, we explored the delivery of exosomal miR-663b in cervical cancer. We measured the level of miR-663b in exosomes purified from H8 cervical precancerous cells and HeLa, CaSki, and SiHa cervical cancer cells. The results showed that CaSki cells, which were selected for further research, secreted exosomes containing the highest amount of miR-663b (Fig. 3A), while the content of miR-663b in HeLa and SiHa exosomes is lower than that in CaSki. Exosomes derived from CaSki cells were isolated by ultracentrifugation and confirmed to have a typical biconcave disc shape by transmission electron microscopy (TEM) (Fig. 3B), and the positive markers (TSG101, CD63 and CD81) of exosomal proteins were measured by WB (Fig. 3C). NTA proved that the concentration and size of the isolated exosomes were consistent with previous reports [9] (Fig. 3D). Subsequently, we incubated HUVECs with DAPI and PHK67-labeled CaSki exosomes for 0 h, 2 h, 4 h and 8 h, and a green fluorescent punctate signal inside the cytoplasm of recipient HUVECs indicated the internalization of exosomes (Fig. 3E).

To further verify that exosomes can transport miR-663b into target cells, we used lentivirus and the corresponding scrambled control virus to infect CaSki cells and construct stable transgenic strains. Then, exosomes collected from different groups (Exo-miR-663b up, Exo-miR-663b inh, Exo-up NC and Exo-inh NC) were added to HUVECs for incubation. Compared with the control group, the level of miR-663b in HUVECs transfected with miR-663b-upregulated exosomes was significantly increased, while the level of miR-663b in HUVECs transfected with miR-663b-transfected exosomes was lower than that in the NC group (Fig. 3F). We also measured the expression level of VCL in each group. In contrast with the expression of miR-663b, the expression of VCL was significantly reduced in the Exo-miR-663b up group and increased in the Exo-miR-663b inh group (Fig. 3G). Our results proved that exosomal miR-663b secreted by CaSki cells can be delivered into HUVECs and inhibit VCL expression.

### Exosomal miR-663b promotes angiogenesis in-vitro

**Fig. 4:**
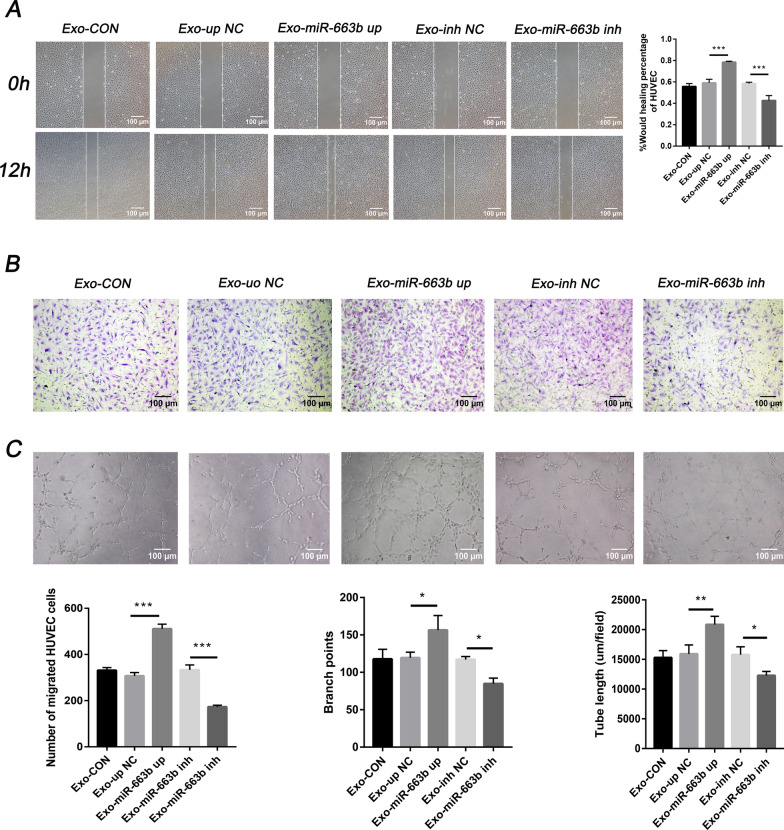
Exosomal miR-663b promotes angiogenesis in-vitro. **A** A wound healing assay was performed to detect the migratory ability of HUVECs after treatment with CaSki exosomes, miR-663b-up exosomes, miR-663b-inhibitor exosomes and NC exosomes (magnification ×100). **B** Transwell assays were performed to detect the effect of HUVECs after treatment with CaSki exosomes, miR-663b-up exosomes, miR-663b-inhibitor exosomes and NC exosomes (magnification ×100). **C** An in-vitro Matrigel tube formation assay was performed to evaluate the angiogenic ability of HUVECs after treatment with CaSki exosomes, miR-663b-up exosomes, miR-663b-inhibitor exosomes and NC exosomes (magnification ×100). The number of branches and the tube length per field were analyzed. **P < 0.01 and ***P < 0.001. VCL, vinculin

To determine the in-vitro role of exosomal miR-663b in angiogenesis, we added exosomes from different treatment groups of CaSki cells to HUVECs and measured the functions of wound healing, migration and tube formation. The results showed that the wound healing and migration capabilities of HUVECs in the Exo-miR-663b up group were stronger than those in the Exo-up NC group. Additionally, HUVECs cocultured with Exo-miR-663b inh exosomes had weakened wound healing and migration capabilities compared with the Exo-inh NC group (Fig. 4A, B). As predicted, the tube formation rate of HUVECs in the Exo-miR-663b up group was higher than that in the control group and untreated group (CON). Moreover, the tube formation rate of HUVECs cocultured with exosomes in the Exo-miR-663b inh group was lower than that in the control and untreated groups (Fig. 4C). These results proved that miR-663b from CaSki cell exosomes contributes to HUVEC migration and tube formation and can promote angiogenesis in-vitro.

### Exosomal miR-663b promotes angiogenesis in-vivo

**Fig. 5:**
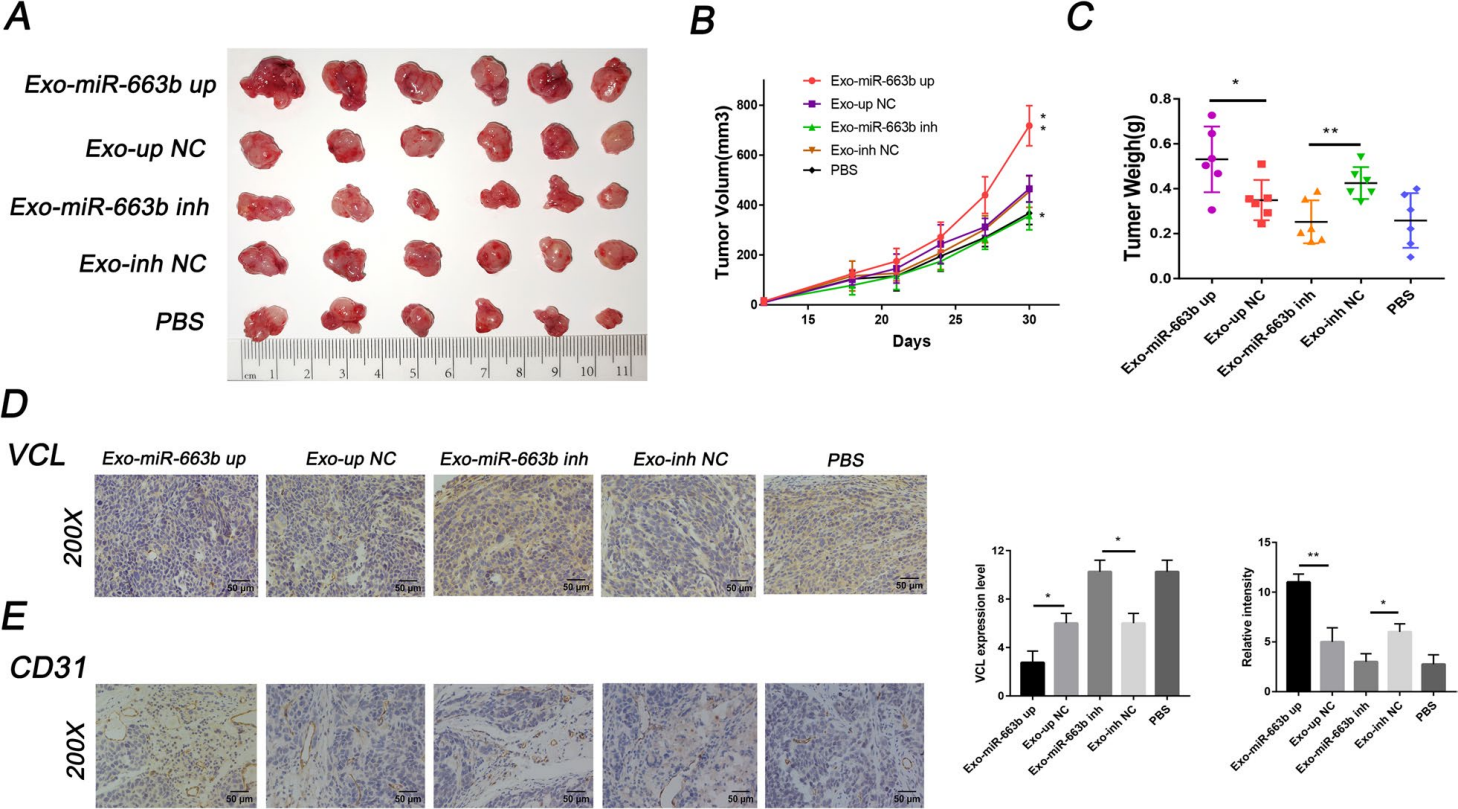
Exosomal miR-663b promotes angiogenesis in-vivo. **A** Comparison of the tumors (CaSki) taken from the Exo-miR-663b up, Exo-up NC, Exo-miR-663b inh, Exo-inh NC groups. An equal volume of PBS was injected as a blank control. **B** Growth curves of tumors were generated by measuring tumor volumes every three days (n = 6). **C** Analysis of tumor weight in each group (n = 6). **D** IHC was performed to measure the expression level of VCL in tumors (CaSki) taken from the Exo-miR-663b up, Exo-up NC, Exo-miR-663b inh, Exo-inh NC groups. **E** IHC was performed to demonstrate the existence of endothelial tissue and to assess tumor angiogenesis using a CD31 antibody. *P < 0.05, **P < 0.01 and ***P < 0.001. VCL, vinculin

We subsequently used a mouse model of tumor implantation to evaluate the effect of exosomal miR-663b on angiogenesis in-vivo. CaSki cells (5 × 10^6^) were injected subcutaneously into the armpits of female nude mice to form tumors. When the tumor size reached 50 mm^3^, exosomes from the miR-663b up, miR-663b inh, up NC and inh NC groups were randomly injected into the center of the xenograft tumor (n = 6/group), and the untreated group was replaced with PBS. The results showed that compared with the control group, the diameter and weight of the tumors in the Exo-miR-663b up group were significantly increased, while those in the Exo-miR-663b inh group were significantly reduced (Fig. 5A, B). IHC was performed to evaluate the role of miR-663b in tumor tube formation and showed that the overexpression of miR-663b resulted in increased blood vessel density, while the miR-663b inhibitor significantly inhibited tube formation (Fig. 5C). The expression of VCL was then measured by IHC, and the results showed that VCL increased in the Exo-miR-663b inh group and decreased in the Exo-miR-663b up group (Fig. 5D) and had a negative relationship with tumor blood vessel density (Fig. 5E). Therefore, we conclude that miR-663b, as a carcinogen, can be delivered into target cells through exosomes to downregulate VCL expression, thereby promoting tumor growth and angiogenesis in-vivo.

### Identification of VCL in angiogenesis

**Fig. 6:**
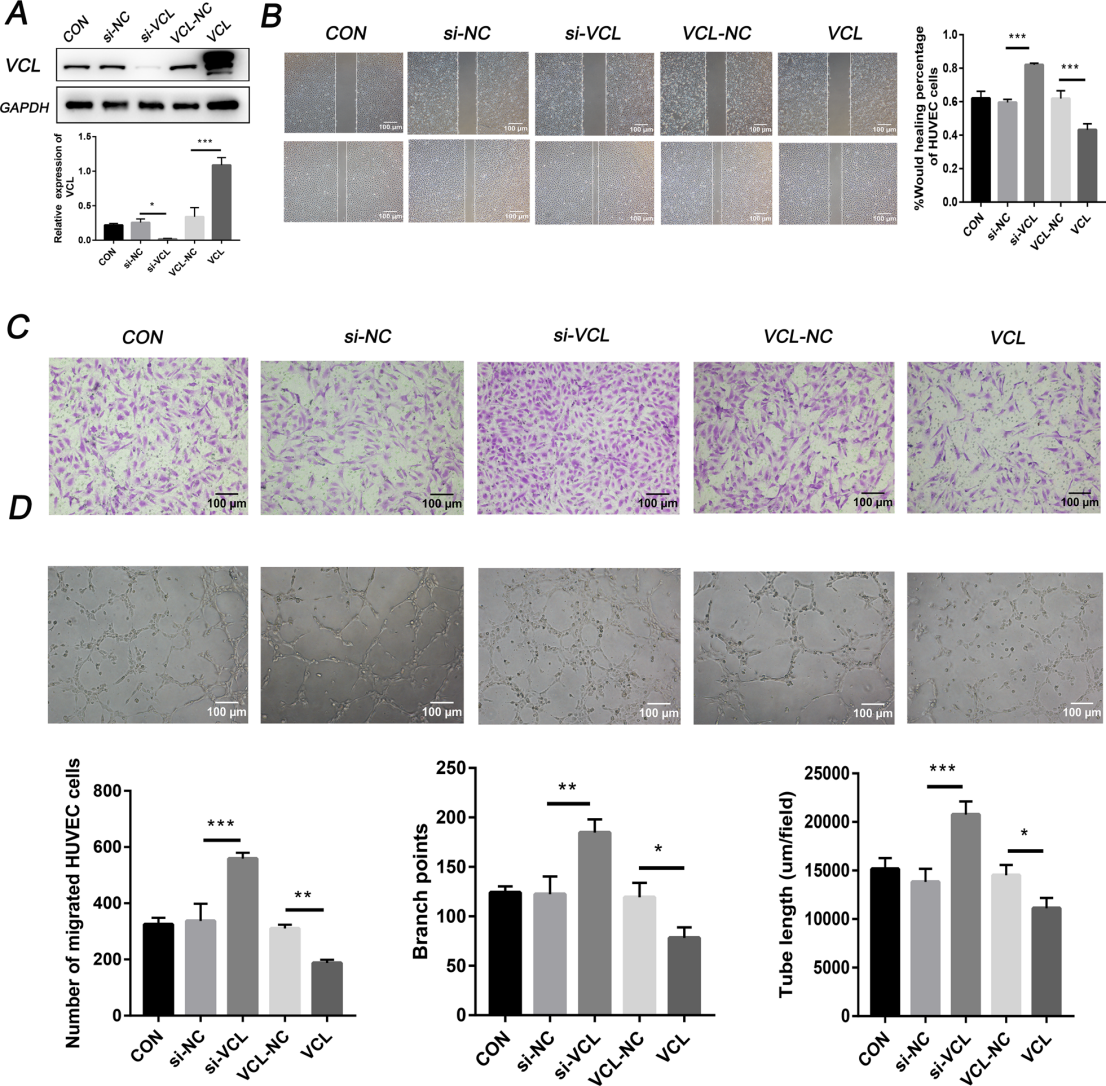
Identification of VCL in angiogenesis. **A** The protein levels of VCL were determined by Western blot analysis in HUVECs from the CON, si-NC, si-VCL, VCL-NC and VCL groups, as well as quantification of protein expression levels. **B** A wound healing assay was performed to detect the migratory ability of HUVECs from the CON, si-NC, si-VCL, VCL-NC and VCL groups (magnification ×100). **C** Transwell assays were performed to detect the effect of HUVECs from the CON, si-NC, si-VCL, VCL-NC and VCL groups (magnification ×100). **D** An in-vitro Matrigel tube formation assay was performed to evaluate the angiogenic ability of HUVECs from the CON, si-NC, si-VCL, VCL-NC and VCL groups (magnification ×100). The number of branches and the tube length per field were analyzed. *P < 0.05, **P < 0.01 and ***P < 0.001. VCL, vinculin

**Fig. 7:**
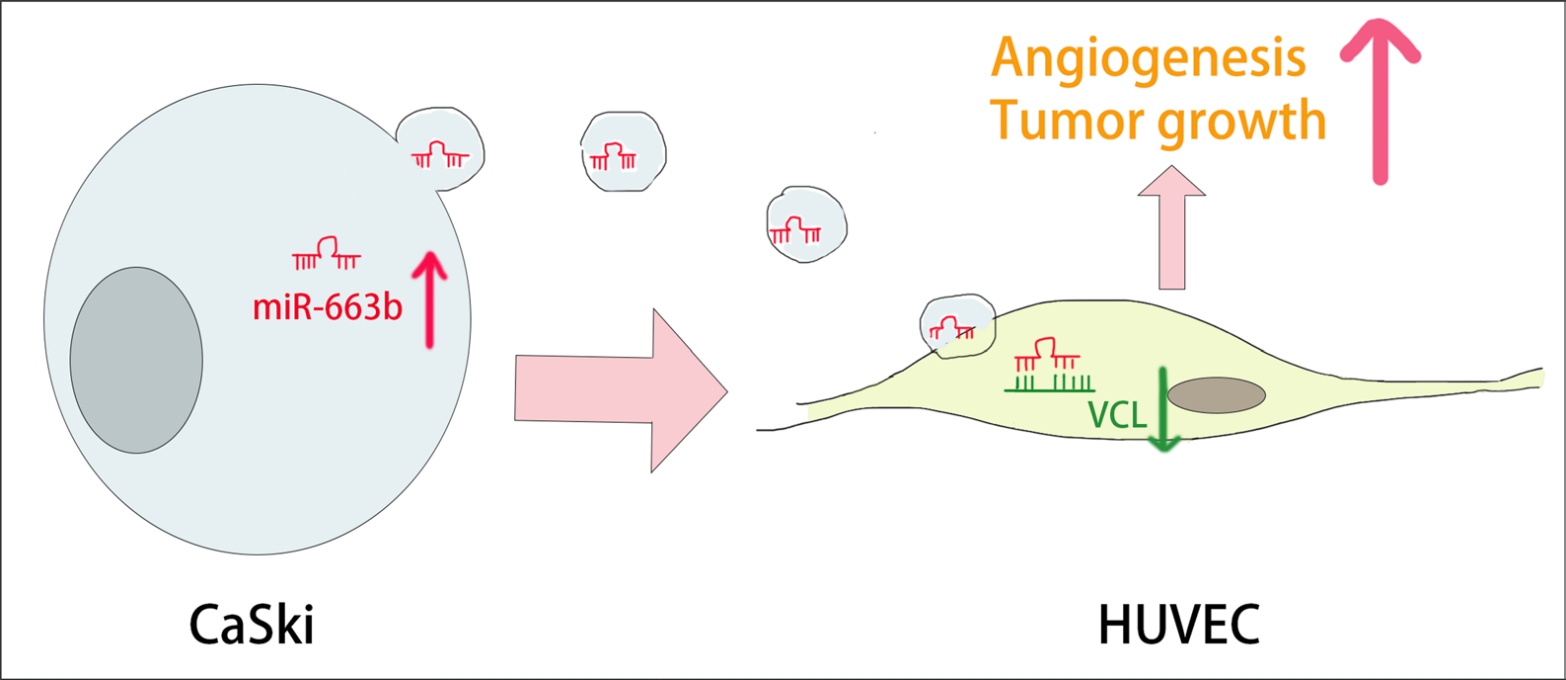
Schematic representation of the miR-663b/VCL axis in cervical cancer angiogenesis

To explore whether VCL regulates the migration and tube formation of HUVECs, we used lentiviral vectors to overexpress VCL in HUVECs. After 4 weeks of cell transfection and puromycin selection, a stable VCL overexpression strain was constructed. Conversely, HUVECs were transfected with si-VCL or control siRNA to inhibit the expression of VCL. WB was used to verify the efficiency of VCL gene overexpression and knockdown (Fig. 6A). The results of a wound healing assay, Transwell assay and tube formation experiments showed that compared with the respective control groups, overexpression of VCL significantly inhibited the migration and tube function ability of HUVECs, while knocking down VCL induced the enhancement of HUVEC migration and tube function (Fig. 6B–D). The underlying mechanism of the miR-663b/VCL axis in cervical cancer angiogenesis is shown in Fig. 7.

## Discussion

Metastasis is an important biological behavior of tumor cells that is closely related to poor prognosis and seriously threatens the health and life of patients. A variety of complex factors affect tumor metastasis [29], among which angiogenesis makes a notable contribution [30, 31], so it is hopeful that angiogenesis will become a therapeutic target for advanced cervical cancer [7, 32]. However, currently available antiangiogenic therapies are relatively limited, and the mechanism of angiogenesis is still unclear. Cancer-derived exosomes participate in the construction of the tumor microenvironment to promote tumorigenesis, progression, migration and invasion [33, 34]. Previous studies have shown that exosomes play a key role in promoting the proliferation of tumor cells, helping cancer cells escape the immune system or the elimination of drugs, and creating a favorable microenvironment for the spread of cancer cells [35–37]. In recent years, discoveries regarding the influence of exosomes on tumor angiogenesis have gradually been reported [39], which suggests that the specific mechanism of cancer-derived exosomes in cervical cancer angiogenesis may be a new prospect for exosome research.

Exosomes encapsulate a variety of biologically active substances, including miRNAs, and transport them to new target cells to mediate intercellular communication [8]. miRNAs are small noncoding RNAs with a length of 17–24 nt. By binding to the 3′-UTR of target gene mRNA completely or incompletely, miRNA can cause mRNA degradation or posttranscriptional inhibition, thereby regulating cell proliferation, differentiation, migration, disease occurrence and progression [40, 41]. It was reported that miR-663b plays a role as a cancer-promoting factor in the progression of colorectal cancer [42], nasopharyngeal carcinoma [43], endometrial carcinoma [44], osteosarcoma [46], bladder cancer [45] and other cancers, but its role in cervical cancer has not been studied. In our current study, we found that miR-663b was upregulated in cervical cancer patients and exosomes secreted by cervical cancer cell lines. Kaplan–Meier plotter analysis showed that the expression of miR-663b was associated with poor prognosis of cervical cancer. Moreover, miR-663b derived from CaSki cell exosomes could be internalized by HUVECs and significantly promote their migration and tube formation ability. Above all, these results suggested that miR-663b was involved in the molecular mechanism of cervical cancer angiogenesis.

According to the results predicted by online bioinformatics analysis, miR-663b can bind to the 3′-UTR of VCL mRNA with a high priority. The negative regulatory relationship between VCL and miR-663b was verified by dual luciferase reporter assay and directly detected in clinical samples. VCL is a widely expressed actin-binding protein that plays an essential role in focal adhesion formation, cell proliferation and regulation of the actin cytoskeleton [17–20]. It was reported that the expression of VCL was reduced in a variety of cancers and was involved in the invasion, metastasis and apoptosis of tumor cells [24–27]. Early studies also found that fibroblasts isolated from VCL-deficient mice showed decreased adhesion strength and increased migration ability, while VCL-null cells showed the opposite result [47]. In addition, the expression of VCL in highly metastatic colorectal cancer (CRC) cell lines and metastatic tissues was significantly downregulated and became an independent prognostic factor for CRC [21]. Recent studies have found that the absence of VCL expression in squamous epithelial tumors was related to the metastatic potential of tumors [18, 22, 48], and the overexpression of VCL in cancer cells can inhibit tumorigenic and metaplastic ability [49]. However, few studies have explored the biological role of VCL in cervical cancer metastasis and angiogenesis. Our results primarily revealed that the expression of VCL was reduced in cervical cancer tissues and was associated with tumor metastasis. Exosomes secreted from CaSki cells could transport miR-663b to HUVECs to inhibit VCL expression. The overexpression of VCL significantly inhibited the migration and tube function of HUVECs, while knockdown of VCL caused HUVEC migration and tube function enhancement. Animal studies have shown that adding miR-663b-containing exosomes to tumor tissues can reduce the expression of VCL protein and weaken angiogenesis, thereby inhibiting the growth of transplanted tumors in mice. IHC analysis also shows a negative correlation between VCL and angiogenesis. Our results indicated that VCL could inhibit cervical cancer angiogenesis both in-vivo and in-vitro.

In our current study, one of the limitations is that the limited clinical sample size prevents us from performing survival analysis of VCL in cervical cancer. In addition, experiments on whether there is an interaction between VCL and the angiogenesis-targeted drug Bevacizumab were not carried out, also the signaling pathways involved in miR-663b/VCL axis in cervical cancer angiogenesis need further research.

## Conclusion

Considering the key position of angiogenesis in tumorigenesis and progression, exploring new antivascular targets has become a primary task. Our research innovatively found that exosomes secreted by CaSki cells could carry miR-663b to HUVECs to promote cervical cancer angiogenesis by inhibiting the expression of VCL. These findings implied that inhibiting the expression of miR-663b-enriched exosomes or blocking their delivery may be a new strategy for antiangiogenic therapy in cervical cancer. In our future research, we will focus on exploring whether miR-663b delivered by exosomes can be effectively applied to patients with cervical cancer to become a new diagnostic biomarker for cancer patients and an antiangiogenesis therapeutic target.

## Data Availability

All data used in this study can be obtained from the corresponding author upon reasonable request.
